# Innovative Method Using Adhesive Force for Surface Micromachining of Carbon Nanowall

**DOI:** 10.3390/nano10101978

**Published:** 2020-10-06

**Authors:** Hyeokjoo Choi, Seokhun Kwon, Seokwon Lee, Yonghyeon Kim, Hyunil Kang, Jung Hyun Kim, Wonseok Choi

**Affiliations:** 1 Department of Electrical Engineering, Hanbat National University, Daejeon 34158, Korea; hyukju1210@hanmail.net (H.C.); kwon1567@naver.com (S.K.); dltjrdnjs000@naver.com (S.L.); hikang@hanbat.ac.kr (H.K.); 2 Department of Advanced Materials Engineering, Hanbat National University, Daejeon 34158, Korea; kjw4493@naver.com (Y.K.); jhkim2011@hanbat.ac.kr (J.H.K.)

**Keywords:** carbon nanowall, sonication, surface micromachining, surface modification

## Abstract

The application of a carbon nanowall (CNW) via transfer is very demanding due to the unusual structure of vertically grown wall-shaped that easily collapses. In addition, direct growth on a device cannot obtain a precision-patterned shape because of the temperature limit of the photoresist (PR). Therefore, in this paper, we demonstrate a new CNW surface micromachining technology capable of direct growth. In order to reduce unexpected damage caused by chemical etching, a physical force was used to etch with the adhesive properties of CNWs that have low adhesion to silicon wafer. To prevent compositing with PR, the CNW was surface modified using oxygen plasma. Since there is a risk of surface-modified CNW (SMCNW) collapse in an ultrasonic treatment, which is a physical force, the CNW was coated with PR. After etching the SMCNW grown on PR uncoated area, PR was lifted off using an acetone solution. The effect on the SMCNW by the lift-off process was investigated. The surface, chemical, and structural properties of PR-removed SMCNW and pristine-SMCNW were compared and showed a minimal difference. Therefore, the CNW surface micromachining technique was considered successful.

## 1. Introduction

Graphite-related materials, such as graphene, carbon nanotubes (CNTs), and carbon nanowalls (CNWs) have continuously attracted interest in many fields [1,2,3,4,5,6,7,8,9,10]. A wall-shaped CNW is based on graphene sheets standing vertically, to which electrons or desired materials can attach to on both sides because CNWs have a lamella structure and the largest reaction area among these materials. Furthermore, CNW, unlike graphene and CNTs, does not require a metal catalyst, such as copper [11], nickel [12], gold [13], or platinum [14], during the growth process. At a relatively low temperature, compared to other graphite-related materials, a catalyst-free CNW is grown using various chemical vapor deposition (CVD) techniques or a sputtering system [15,16,17,18,19].

The CVD method offers easy to synthesize thin-films, such as silicon-based (SiO_2_, SiN*_x_*, SiO*_x_*N*_y_*, a-Si, etc.) and carbon thin-films with high purity materials that have good stability and uniformity. In the synthesis mechanism of CNW via CVD, carbon radicals separate from the injected source gas in the chamber and are adsorbed on the surface of the substrate to create several layers of graphene, and the wall grows vertically at the graphene’s defect site [20,21]. In the case of branches that grow beside the nanowalls of CNW, they grow according to subtle variations in temperature and source power [22,23].

With regard to the growth of graphite-related materials through the CVD method, which requires a high temperature, directly growing on a desired area in the photolithography process from the photoresist (PR) temperature poses a limitation. Therefore, graphite-related materials have been grown on other substrates and then transferred to the wanted layer via microfabrication [24]. Since wall-shaped CNWs have a risk of collapse, its transfer is very demanding and, to the best of the authors’ knowledge, very little is known about CNW micromachining techniques. Accordingly, there are limitations in terms of application and requires further studies because CNWs cannot selectively grow in the patterned area with precision.

In this work, surface micromachining of a CNW through surface modification and physical force without chemical etching is suggested. A CNW was grown on the substrate via microwave-plasma-enhanced CVD (MPECVD) with a mixture of methane (CH_4_) and argon (Ar) gases at high temperatures (600 °C). When a non-polar CNW is coated with a non-polar PR, the two are very strongly attracted to each other, and the PR is not completely removed in the development process after exposure. To resolve this problem, the CNW was surface modified under oxygen (O_2_) plasma to lower the strong adhesion between the PR and the CNW by a surface polarity change. The required area was coated with the PR, and the undesired region was removed through sonication in deionized (DI) water. After removing the PR using acetone, the surface, structural, and chemical changes of the surface-modified CNWs (SMCNW) were observed. It was confirmed whether the CNW remained in the area that had been removed through sonication.

## 2. Materials and Methods

### 2.1. Reagents and Materials

Silicon (Si) wafers (100, *p*-type) were purchased from Taewon Scientific Co., Ltd. (iTASCO, Seoul, Korea). Trichloroethylene (≥99.5%), acetone (≥99.5%), and methanol (≥99.8%) solutions were purchased from Sigma-Aldrich. DI water (≥18.2 MΩ∙cm @25 °C) was obtained using a water purification system (YOUNG IN Chromass Co., Ltd., Anyang-si, Korea, aquapuri 5 series). Silicon dioxide (SiO_2_, 99.999%) and silver (Ag, 99.99%) targets were purchased from Taewon Scientific Co., Ltd. (iTASCO, Seoul, Korea). PR (AZ GXR-601 46cp) and its developer (AZ 300 MIF) were purchased from Sigma-Aldrich (St. Louis, MO, USA).

### 2.2. Preparing the Substrates and Deposition of Adhesion-Increased Layer

To clean the surface of a 4-inch Si wafer, which was the starting material, ultrasonic cleaning was performed using trichloroethylene, acetone, methanol, and DI water, in that order, for 10 min each. The SiO_2_ and Ag layers were deposited using a radio frequency (RF) magnetron sputtering system under different conditions using their 4-inch targets (Table 1). In the patterned area where the CNW should have remained, a SiO_2_ layer, which was used as an insulator, was first deposited, followed by Ag. The Ag layer, a transition metal, was used to improve the adhesion between the CNW and its under layer.

### 2.3. Growth of CNW and Its Surface Modification

After the prepared Si wafer was placed on the stage inside the MPECVD (ASTeX-type, Woosin CryoVac, Uiwang-si, Korea, 2.45 GHz microwave) chamber, a 10^−4^ Torr base vacuum was maintained. To form a plasma ball on the substrate producing 1300 W of microwave power, 20 sccm CH_4_ and Ar, respectively, were injected into the chamber. The CNW was subsequently grown on the entire substrate for 10 min in a working vacuum of 10^−2^ Torr and at a 600 °C stage temperature. After CNW growth completion, the CNW was surface modified via plasma treatment, by injecting 20 sccm of O_2_ gas with 200 W RF power for 2 min. The SMCNW was etched with the nanowall branches and edge parts, and was polarized through attachment of the functional group.

### 2.4. Innovative Method of Top-Down Surface Micromachining of CNW Based on Its Adhesive Properties

**Figure 1:**
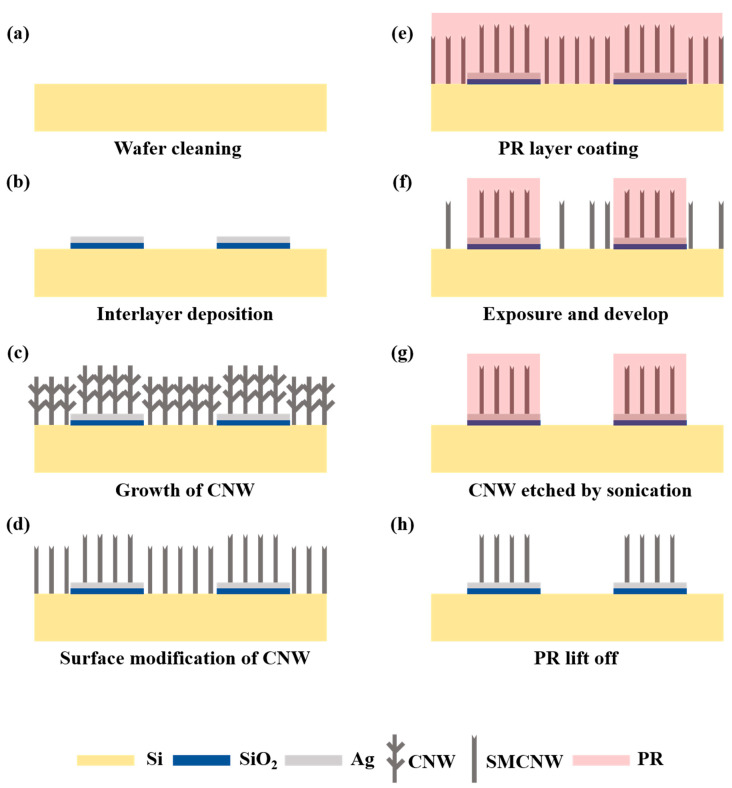
Fabrication process of top-down surface micromachining in surface-modified carbon nanowall. (**a**) Wafer cleaning process; (**b**) Patterning and deposition of Ag/SiO_2_ layer; (**c**) Growth of CNW via MPECVD; (**d**) Surface modification of CNW by O_2_ plasma; (**e**) Coating the PR layer on the sample; (**f**) Exposure and develop process; (**g**) Surface micromachining of SMCNW by sonication; (**h**) PR lift-off process.

Carbon etching techniques using oxidation have been extensively studied [25,26]. If the metal used as an electrode, however, is unintentionally oxidized in the application, such as a sensor, the energy band gap of the metal electrode employed by the metal oxide layer may increase, causing the sensor’s sensitivity to decrease. Therefore, an etching technique using physical force that does not employ a chemical etchant and using the CNW’s adhesive properties was devised. A PR layer was used for coating the sample on which the SMCNW was grown. The PR we applied was a mixture of diazonaphthoquinone sulfonic ester and cresol novolak resin. Therefore, it can be inferred that the PR we used is a *p*-type that dissolves easily in a base due to its solubility variation in the UV absorbed part. The PR was coated on a flat material and it was spin-coated to have a height of about 3 μm. After the PR-coated sample was exposed to a 180 mJ UV light as part of the development process, the exposed PR area was dissolved using its developer. Because the adhesion between the CNW and the Si wafer was feeble, more than half of the undesired area of the CNW was dissolved along with the PR during the development process. The remaining CNW that needed to be removed was placed in DI water and was sonicated for 10 min. To prevent deformation of the PR, the SMCNW was etched in minimal light. The CNWs in all the unwanted areas that had been etched were immersed in an acetone solution to remove the PR. After the PRs were completely removed, the CNWs were soaked in DI water and were immediately taken out and then dried naturally at room temperature. Figure 1 shows the overall fabrication process of SMCNW surface micromachining.

### 2.5. Characterization and Measurement of the Samples

By confirming the C atoms by energy-dispersive X-ray spectroscopy (EDS) analysis, it was demonstrated whether the residual of the SMCNW was etched completely by the physical force. The contact angle, which was averaged for 5 s immediately after the drop, was observed by dropping 3 μL DI water, a polar molecule, on the sample to check whether the CNW and SMCNW were polar or non-polar. The differences before and after surface micromachining of the SMCNW grown in the desired area were compared in terms of their surface, structural, and chemical properties. The surface characteristics were determined via field emission scanning electron microscopy (FESEM, HITACHI, Tokyo, Japan, S-4800) images and atomic force microscopy (AFM, Suwon-si, Korea, Park systems, XE-100; scanning size: 2 μm × 2 μm). Raman spectroscopy (NOST, Seongnam-si, Korea, FEX; excitation wavelength: ~531 nm; excitation power: ~0.3 mW) was conducted to observe the structural characteristics of the samples. From the Raman spectrum, inherent peaks of the graphite-related materials, and assorted information, such as the defects, the thickness of the graphene layers, and the distinctive structural properties of the CNW, were obtained. The chemical properties of the samples were investigated using X-ray photoelectron spectroscopy (XPS, ThermoFisher Scientific, Waltham, MA, USA, K-Alpha+) analysis, which showed the chemical composition and the 1–10 nm bonding thickness of the surface.

## 3. Results and Discussion

### 3.1. Application of the CNW Photolithography Process

**Figure 2:**
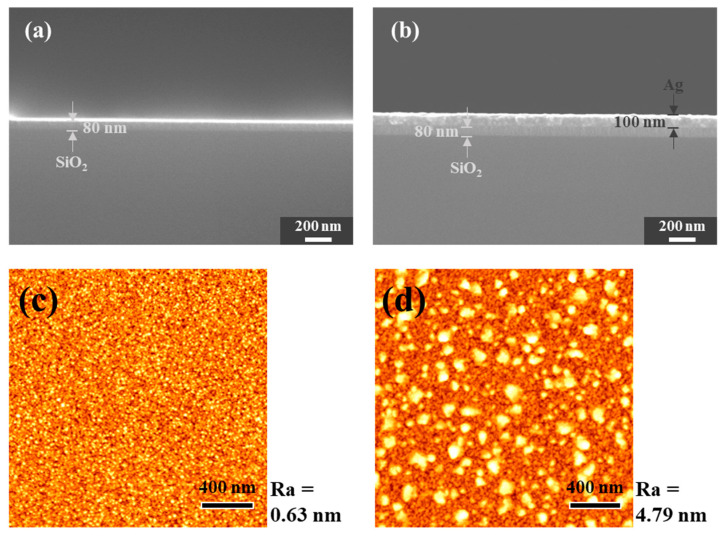
FESEM cross-section image of (**a**) SiO_2_ and (**b**) Ag/SiO_2_ deposited Si wafer; atomic force microscopy (AFM) 2D image of (**c**) SiO_2_ and (**d**) Ag/SiO_2_ layer.

Figure 2 shows the surface morphology of SiO_2_ and Ag/SiO_2_ deposited on the cleaned Si wafer. Figure 2a,b shows the thickness of the SiO_2_ layer and the Ag layer, respectively, with the Ag/SiO_2_ layer being about 180 nm thick. In Figure 2c, the average roughness (Ra) of the SiO_2_ layer is 0.63 nm. The skewness of the SiO_2_ layer is −0.473, which is a negative value, so it has an upwardly skewed roughness. Figure 2d shows the surface roughness of the Ag/SiO_2_ layer, while Ra is 4.788, which has a rougher surface than the SiO_2_ layer. The skewness of Ag/SiO_2_ is −1.333, which has an upwardly skewed roughness.

**Figure 3:**
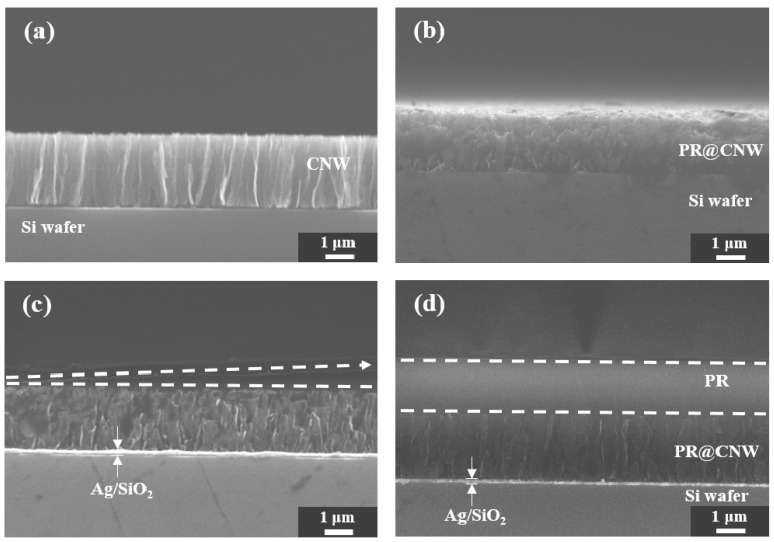
FESEM cross-sectional image of the (**a**) carbon nanowall (CNW), (**b**) photoresist (PR)@CNW, and (**c**) PR@CNW between the desired and non-removed areas, and the (**d**) PR-coated CNW.

Positive PR was patterned for CNW surface micromachining. Figure 3a shows a FESEM cross-sectional image of the CNW grown on a Ag/SiO_2_ layer. The height of the CNW grown on the Ag/SiO_2_ layer was about 1.7 μm, and the height of the CNW composited with a PR (PR@CNW) in Figure 3b was about 1.9 μm. PR@CNW material was obtained by coating PR on CNW without surface modification, exposing it to UV light, and dissolving PR in a developer. However, during the development process, the PR area exposed to light was hardly dissolved in the developer and was not removed easily, even after sonicating in DI water. Through the comparison of the PR@CNW and CNW images, the CNW and PR were bonded with strong adhesion even after the development process. Figure 3c confirms that the unexposed area retained its pyramid shape due to the characteristics of the positive PR. In addition, as shown in Figure 3d, even when the wall-shaped CNW was coated with a PR, it was uniformly applied with a thickness of about 4 μm. It was spin-coated in a flat material so that the PR was about 3 μm; however, when PR was coated onto CNW, PR@CNW was formed and the height was 4 μm.

**Figure 4:**
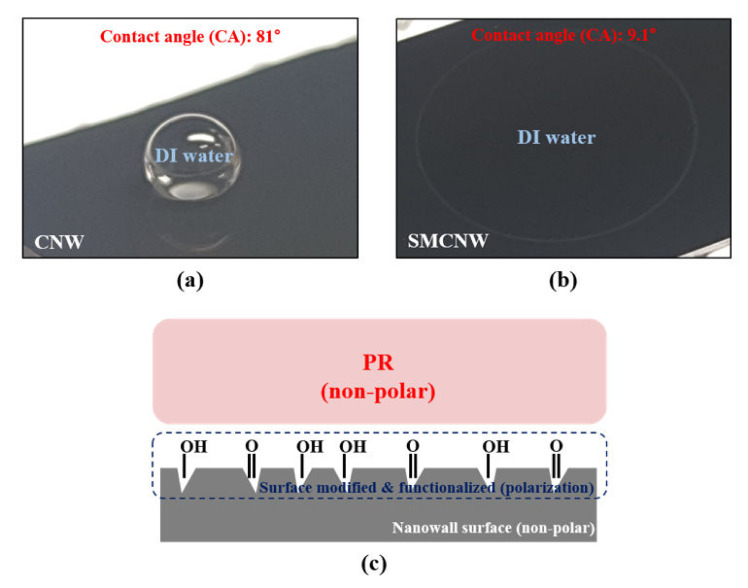
Contact angle of the (**a**) CNW and (**b**) surface-modified carbon nanowall (SMCNW). (**c**) Schematic of the positive PR-coated SMCNW.

To determine the reason for the strong bond between the PR and the CNW, the contact angle between the DI water and the CNW was confirmed, as shown in Figure 4a. Water molecules are polar and do not mix well with non-polar molecules like oil. The contact angle of the CNW was 81°, which means that it was hydrophobic and non-polar. On the contrary, the SMCNW in Figure 4b has a low contact angle with DI water due to activation of the functional group on the nanowall surface, and is hydrophilic and polar. The extreme change in the contact angle through surface modification of the nanowall can also be explained by the Laplace pressure, which results from the difference in surface energy between the nanowall and the buffer layer [27,28]. The PR that was used in this study was a non-polar molecule. As a result, the mixing of non-polar molecules caused strong adhesion, enabling the PR@CNW to remain even after the development process. Figure 4c explains why SMCNW has low adhesion to PR, unlike CNW. As with the polar molecular formation on the surface, which is the reason for carrying out the dehydration baking process, SMCNW was employed to reduce the adhesion between the CNW and the PR.

**Figure 5:**
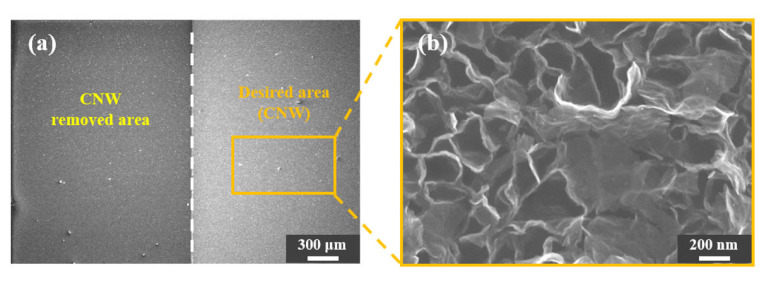
FESEM surface image (**a**) after sonication of the CNW grown on a Si wafer (removed area) and a Ag/SiO_2_ layer (desired area) without PR coating. (**b**) FESEM image of 300 K magnification in the CNW grown on the desired area after sonication.

An attempt was made to perform surface micromachining using the adhesion of the Ag/SiO_2_ layer and CNW, without using a PR. Figure 5 shows a FESEM image after sonication of a CNW-grown sample in DI water without coating it with a PR. As the CNW grown directly on the Si wafer dissociated, the boundary with the CNW grown on the Ag/SiO_2_ layer appeared as shown in Figure 5a. The deformed CNW was observed after sonication, as shown in Figure 5b, to see if the boundary line was quite visible but had an effect on the CNW. As expected, some nanowalls had collapsed and were inappropriate for use with CNW surface micromachining that only employed adhesion.

### 3.2. Observation of the Variation in the SMCNW to which Surface Micromachining Technology Was Applied via Physical Force

**Figure 6:**
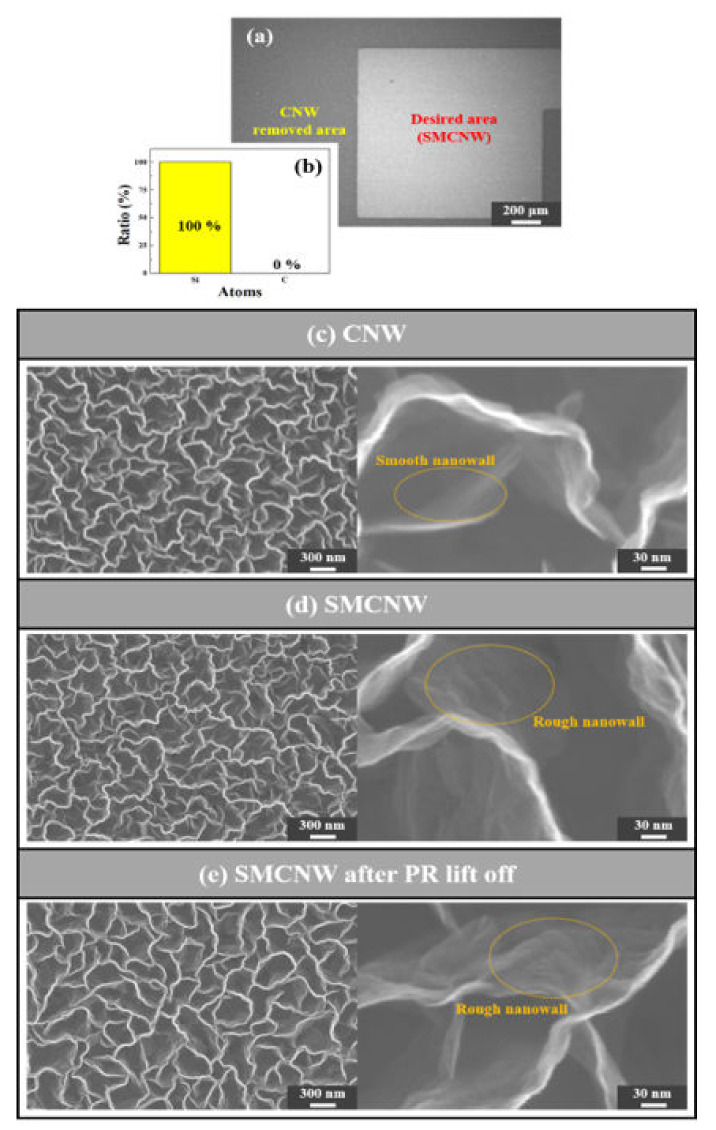
(**a**) FESEM surface image after applying the top-down surface micromachining technology to the SMCNW. (**b**) EDS analysis of the area where the CNW was removed through ultrasonic cleaning. FESEM surface image of the (**c**) CNW, (**d**) SMCNW, and (**e**) PR-removed SMCNW.

With regard to whether CNW is affected by a PR removal agent and a PR that has not been removed, PR cannot be applied to CNW in surface micromachining. Figure 6 shows whether the surface properties of SMCNW are changed by PR or not. Figure 6a shows that the SMCNW is removed through sonication, along with the boundary between the desired and removed areas. Figure 6b shows that the SMCNW dissociated completely, with only Si atoms appearing. Comparing the CNW in Figure 6c and the SMCNW in Figure 6d grown on the Ag/SiO_2_ layer, it can be seen that the surface of the nanowall was roughened by surface modification. The functional group was attached to the roughened area for polarization to lower the adhesion to the PR, and the SMCNW obtained by lifting off the patterned PR from acetone is shown in Figure 6e. The SMCNW after PR removal did not show any noticeable changes from before PR removal, and the nanowall surface had the same rough appearance.

**Figure 7:**
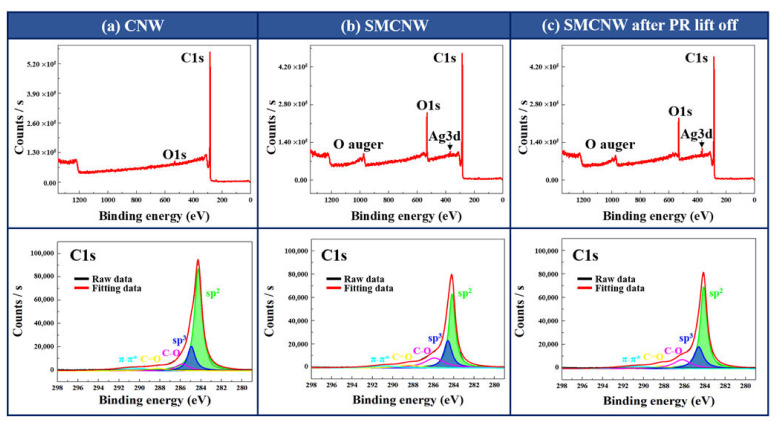
XPS analysis of the (**a**) CNW, (**b**) SMCNW, and (**c**) PR-removed SMCNW.

In CNW chemical bonding, there is sp^3^ bonding in the area where nanowalls are grown from the buffer layer, and since branches are grown from the nanowall, sp^2^ (a graphene layer of CNW) and sp^3^ bonding exist simultaneously [20,21]. These two bonds can be seen on the CNW C1s peak in Figure 7a, and a small O1s peak caused by exposure to air appeared in the XPS survey. In the SMCNW XPS survey (Figure 7b), a Ag3d peak and an O1s peak larger than that of the CNW were identified. It can be inferred that the appearance of a Ag3d peak in the SMCNW XPS survey was due to the etching of the O_2_ plasma in the buffer layer. The O1s on the CNW surface had about 1.45 atomic % while the O1s on the SMCW surface had about 14.3 atomic %. Some shoulder and C–O and C=O peaks were identified in the C1s peak of the SMCNW, causing functional groups to form on the surface. Figure 7c shows the chemical properties of the SMCNW after the PR was removed, as obtained via XPS analysis. There were minimal differences in peak and even in components (O1s: 13.54 atomic %), in Figure 7b, before PR coating. From some O1s component difference between SMCNW and PR-removed SMCNW, it can be inferred that the functional group on SMCNW surface is slightly modified by the remaining acetone solution after reacting with PR because of SMCNW solubility [29]. However, comparing the XPS analyses of their chemical bonding, the difference is meaningless. Therefore, the chemical properties of the PR-coated SMCNW and of the SMCNW from which the PR had been removed using acetone indicate that there were no changes in the chemical properties from before the PR coating.

**Figure 8:**
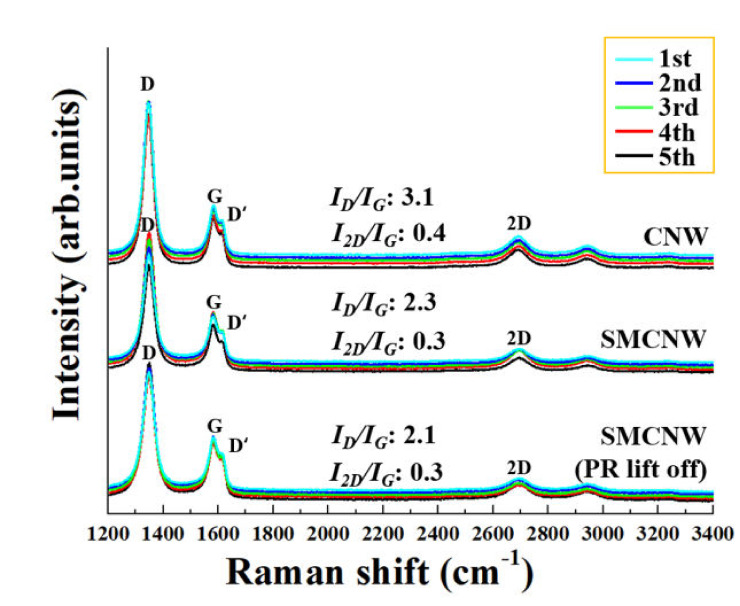
Raman spectra of the CNW, SMCNW, and PR-removed SMCNW.

CNW has unique structural characteristics that differentiate it from other graphite-related materials. Figure 8 shows the Raman spectra of CNW, SMCNW, and PR-removed SMCNW, and the inherent peaks (i.e., D, D’ (edge and defect of graphene), G (sp^2^ bonding), and 2D (thickness of graphene) peaks of graphite-related materials) are equally identified [29,30,31,32,33]. The results obtained through the five trials for each sample (CNW, SMCNW, and PR-removed SMCNW) showed almost constant intensity. The average *I_D_*/*I_G_* (intensity of the D peak/intensity of the G peak) of the SMCNW was smaller than that of the CNW. The bigger the *I_D_*/*I_G_* ratio, the larger the defect. In the XPS analysis result shown in Figure 7, however, the SMCNW should have a higher *I_D_*/*I_G_* ratio than the CNW because the SMCNW was formed from some functional group that was released after the sp^2^ bonding. Nevertheless, the *I_D_*/*I_G_* ratio of the SMCNW was smaller than that of the CNW. It can be inferred that the edge portion of the nanowall branches, which consisted of graphene layers, was reduced through O_2_ plasma etching. The number of graphene layers can be predicted from the *I*_2*D*_/*I_G_* (intensity of the 2D peak/intensity of the G peak) ratio, and the larger the ratio is, the thinner the graphene layers. Therefore, the decrease in the *I*_2*D*_/*I_G_* ratio of the SMCNW can be attributed to the removal of the nanowall branches composed of relatively thin graphene layers compared to the nanowall. The structural properties of the SMCNW with the PR removed were almost identical to those of the SMCNW before PR coating. Therefore, it was demonstrated in this study that structural changes do not occur even after lifting off the PR from the SMCNW.

## 4. Conclusions

Reported in this paper is a new carbon nanowall (CNW) top-down surface micromachining technology utilizing physical force. We propose a technology that grows directly on the substrate in a high temperature (600 °C) rather than using an etchant or transcription. There are three key elements in this method. First, using the fact that CNW has low adhesion with Si wafer and is easily dissociated even when small physical forces are applied; an interlayer (Ag/SiO_2_) that enhances the adhesion between CNW and its under layer was deposited on the desired area. Second, since the non-polar PR and CNW are strongly bonded to each other, the CNW is surface-modified using oxygen plasma to attach functional groups and change its polarity. Third, in order to prevent the collapse of the surface-modified CNW (SMCNW) grown in the desired area due to the physical force, PR is coated and the unnecessary area is etched using sonication. SMCNW grown on Si wafer has low adhesion, so it can be easily removed by sonication and even more than half of it is dissolved along with UV light exposed PR in the developer. It was observed through energy-dispersive X-ray spectroscopy (EDS) analysis that the SMCNW etching site had been completely removed. In order to obtain the reliability of patterned SMCNW through this method, the surface, chemical, and structural properties of PR-removed SMCNW were investigated. All characteristics were not different from the pristine-SMCNW, indicating that surface micromachining was successful. The results obtained from this work can lead to the expansion of the CNW research field, which has not applied micromachining technology, and diversification of its application field.

## Figures and Tables

**Table 1:** Radio frequency (RF) magnetron sputtering condition of SiO_2_ and Ag target.

Parameter	SiO_2_ Target	Ag Target
Substrate temperature	Room temperature	Room temperature
Injection gas	Ar: 34 sccm O_2_: 6 sccm	Ar: 40 sccm
RF power	150 W	150 W
Deposition time	60 min	3 min
Working pressure	1.5 × 10^−2^ Torr	1.5 × 10^−2^ Torr
